# Chinmedomics: a potent tool for the evaluation of traditional Chinese medicine efficacy and identification of its active components

**DOI:** 10.1186/s13020-024-00917-x

**Published:** 2024-03-13

**Authors:** Mengmeng Wang, Fengting Yin, Ling Kong, Le Yang, Hui Sun, Ye Sun, Guangli Yan, Ying Han, Xijun Wang

**Affiliations:** 1 State Key Laboratory of Integration and Innovation of Classical Formula and Modern Chinese Medicines, National Chinmedomics Research Center, National TCM Key Laboratory of Serum Pharmacochemistry, Metabolomics Laboratory, Department of Pharmaceutical Analysis, Heilongjiang University of Chinese Medicine, Heping Road 24, Harbin, 150040 China; 2 State Key Laboratory of Quality Research in Chinese Medicine, Macau University of Science and Technology, Avenida Wai Long, Taipa, Macau China; 3 State Key Laboratory of Dampness Syndrome, The Second Affiliated Hospital Guangzhou University of Chinese Medicine, Dade Road 111, Guangzhou, China

**Keywords:** Chinmedomics, Traditional Chinese medicines, Syndromes/diseases diagnosis, Efficacy evaluation of TCM, Active ingredients discovery

## Abstract

As an important part of medical science, Traditional Chinese Medicine (TCM) attracts much public attention due to its multi-target and multi-pathway characteristics in treating diseases. However, the limitations of traditional research methods pose a dilemma for the evaluation of clinical efficacy, the discovery of active ingredients and the elucidation of the mechanism of action. Therefore, innovative approaches that are in line with the characteristics of TCM theory and clinical practice are urgently needed. Chinmendomics, a newly emerging strategy for evaluating the efficacy of TCM, is proposed. This strategy combines systems biology, serum pharmacochemistry of TCM and bioinformatics to evaluate the efficacy of TCM with a holistic view by accurately identifying syndrome biomarkers and monitoring their complex metabolic processes intervened by TCM, and finding the agents associated with the metabolic course of pharmacodynamic biomarkers by constructing a bioinformatics-based correlation network model to further reveal the interaction between agents and pharmacodynamic targets. In this article, we review the recent progress of Chinmedomics to promote its application in the modernisation and internationalisation of TCM.

## Introduction

Traditional Chinese Medicine (TCM) has become an important part of medical science with a long history and rich clinical experience [1]. Due to its characteristics of fewer side effects, natural growth, and multi-target effects than synthetic drugs, TCM has recently attracted the attention of many medical researchers [2]. It has made prominent contributions to modern medicine, the most successful example being artemisinin extracted from Artemisia annua L. for the treatment of malaria, for which Tu Youyou was awarded the 2015 Nobel Prize in Physiology or Medicine [3]. During these years, herbal formulas have proven effective for patients with COVID-19 in improving cure rates, shortening the course of the disease, delaying disease progression, and reducing mortality [4]. However, despite its significant intervention effect on diseases, the active ingredients and mechanism of most TCM formulas are still unclear due to the lack of methods that are consistent with TCM theory and practice. Thus, the exploration of new scientific strategies, the study of the mechanisms of action of TCM, the scientific interpretation of the connotations of TCM, and the understanding of its biological basis have become urgent clinical challenges.

In recent decades, many research methods have been proposed to reveal the active ingredients and molecular mechanism of TCM against diseases, such as TCM network pharmacology [5], TCM system pharmacology [6], and Fangjiomics [7]. These methods build bridges between herbal compounds and disease targets by searching databases to screen for active compounds. However, whether the selected compounds can be absorbed into serum and exert their bioactivities still needs further verification. In addition, many disease targets have been obtained by previous methods, how to lock core therapeutic targets and key pathways from numerous disease targets has become an important issue [8, 9].

We are introducing an emerging integrated research strategy called Chinmedomics to address these issues [10]. It uses serum pharmacochemistry of TCM to obtain active ingredients, and then further associates these ingredients with identified disease/symptom biomarkers to clarify the pharmacodynamic substance basis and mechanism of action under the premise of effective treatment of diseases. In 2015, Nature commented that Chinmedomics has built a linguistic bridge between TCM and modern medicine, which is of great significance for fully understanding the efficacy of TCM, enhancing the social value of TCM clinical experience, and promoting the academic progress of TCM [11]. At present, Chinmedomics has been widely accepted at home and abroad and has been widely applied in disease/syndrome diagnosis, biomarker discovery, efficacy evaluation, active ingredients discovery, and solving other problems of modern TCM. In the following chapters of this paper, we introduce the application and analytical techniques of Chinmedomics in these areas.

## Background and development of chinmedomics theory formation

Active ingredients in TCM and natural products have long been an interesting research hotspot. Initially, the determination of active ingredients from herbal medicine, such as berberine [12], glycyrrhizic acid [13], tanshinone IIA [14], etc., relies on standardized procedures including extraction, isolation, purification, qualitative analysis, quantitative analysis, derivatization, and pharmacological activity studies [15]. Subsequently, the emergence of new approaches is improving the efficiency of screening for novel functional botanicals. For example, a luciferase-based high-throughput screening (HTS) assay can speed up the drug discovery process by reducing the number of replicate leads [16]. With the continuous deepening understanding of the active ingredients of TCM, it has been found that studying the behavior of herbal ingredients in vivo is a crucial link for screening active ingredients, so in the 1990s, Professor Wang proposed the serum pharmacochemistry of TCM [17–19]. This approach utilizes a combination of high-throughput techniques to analyze and characterize herbal components absorbed into serum, and shows comprehensive superiority in explaining drug changes in vivo and in vitro, as well as drug-drug interactions [20].

The ambiguity of the nature of the disease/syndrome limits the clarification of the mechanism of action of drugs. Fortunately, metabolomics provides an important basis for clarifying the nature of the symptoms and clinical diagnosis of diseases by providing new insights into the pathology of diseases through the confirmation and analysis of biomarkers [21]. For example, using this method, Lv et al. identified and validated 5 metabolites as indicators for the diagnosis of pancreatic cancer, which have higher accuracy and specificity in accurately diagnosing pancreatic cancer than traditional biomarkers [22]. Multifactorial diseases, such as metabolic syndrome and coronary heart disease, are often caused by multiple underlying pathological mechanisms, and advanced metabolomics has helped us gain a comprehensive understanding of the biological processes and pathogenesis of these diseases [23, 24].

**Fig. 1:**
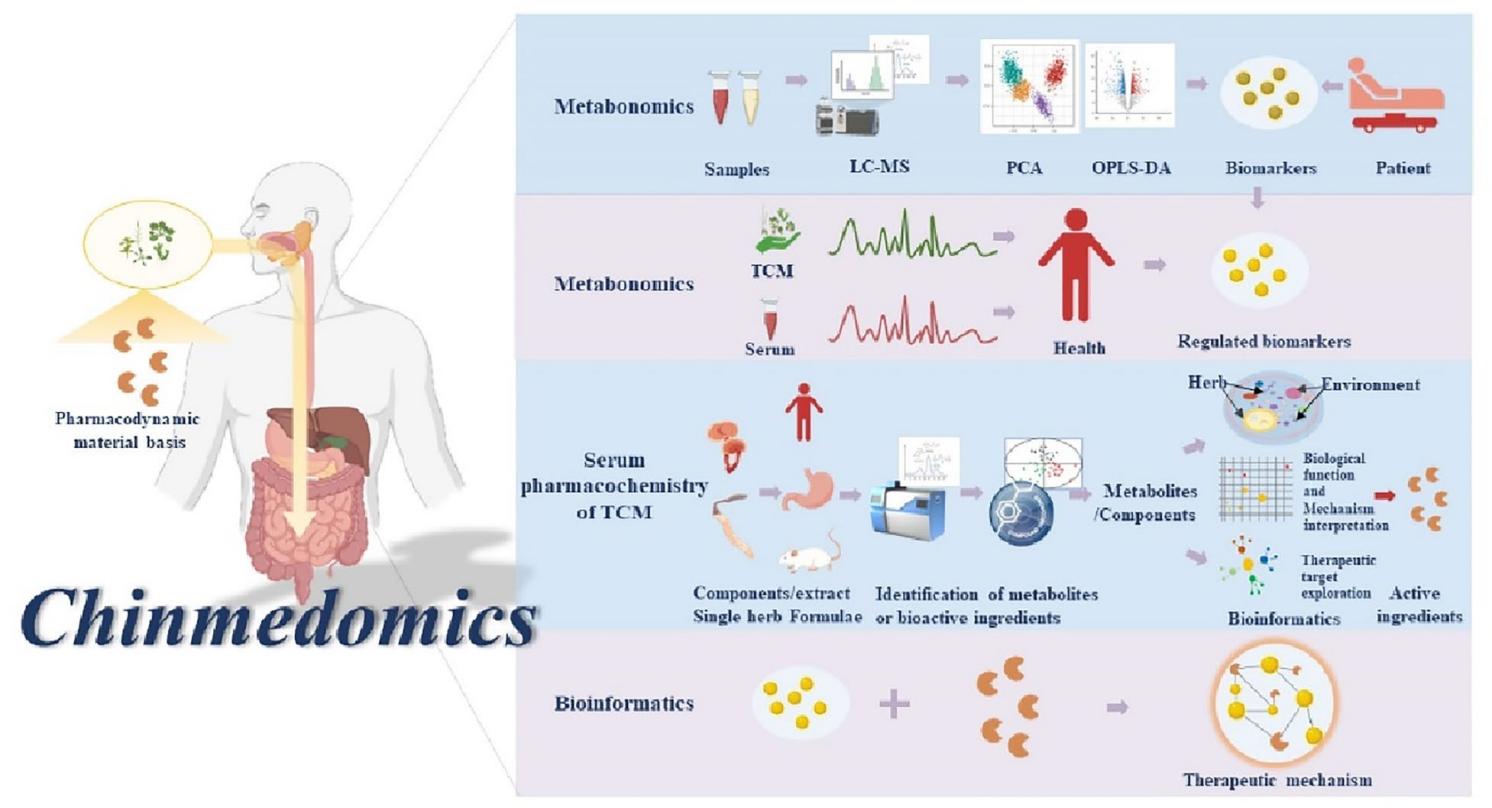
The basic research framework of Chinmedomics

Multidisciplinary integration is essential to fully understand disease and drug mechanisms. Chinmedomics, a new and standardized research approach, has been proposed and established [10]. Based on the metabolomics and serum pharmacochemistry of TCM, this strategy also introduces systems biology, molecular biology and bioinformatics, and forms a multi-omics and multidisciplinary systematic research method to study the efficacy of herbal formulas and discover the active ingredients of TCM. The basic framework is shown in Fig. 1. First, we classify syndromes or diseases according to clinical diagnosis, and use high-throughput, high-sensitivity analysis techniques to analyze the metabolic profiles of metabolites in various biospecimens, comprehensively identify and discover the syndromes/diseases biomarkers and their functions by searching bioinformatics platform and related databases [25, 26]. Second, based on the clinical significance of biomarkers, we accurately analyze disease states and objectively evaluate therapeutic efficacy by analyzing recall biomarkers after intervention with TCM. Thirdly, herbal components are analyzed qualitatively and quantitatively for their absorption and metabolism in serum, and under the premise of ensuring therapeutic efficacy, compounds in TCM are screened for activity through in vitro or in vivo experiments to identify compounds with pharmacological effects, and compounds in TCM are separated, purified, and identified using chromatography, mass spectrometry, and other methods of chemical analysis, and the mechanism of action of TCM on living organisms is further determined through biological experiments, such as gene expression, proteomics etc., to study the mechanism of action of Chinese medicines on living organisms and further determine the bioactive components. Comprehensive characterization of in vivo properties. Finally, a multi-dimensional correlation analysis network model was constructed by mathematical model, which integrated various biomarkers, active ingredients, multiple biochemical factors, and complex clinical phenotypes, so as to elucidate the therapeutic mechanism of active ingredients at different levels. Chinmedomics describes "what happened in TCM ingredients" in vivo, thus it has a more reliable ability to evaluate the efficacy and discover the active ingredients of TCM and is a feasible strategy for the modernization of TCM.

## The analytical technologies of chinmedomics

Chinmedomics applies modern high-throughput and sensitive chromatography-mass spectrometry (LC–MS, GC–MS) and nuclear magnetic resonance (NMR) techniques to characterize herbal constituents and metabolites in various bio-samples, given the complexity and diversity of the biological matrix [27]. In addition, for the structural identification of chemical compounds, the combined strategy of NMR and LC–MS has also been applied [28, 29]. Recently, new mass spectrometry imaging technology has been increasingly utilized to investigate diseases, particularly in the case of cancer [30, 31]. It can visualize the location and quantity of biomarkers in cells and tissues. This allows for the monitoring of biomarker dynamics in disease progression and the observation of molecular interactions between biomarkers and active ingredients of TCM at various stages of the disease [32]. All of these analytical techniques can separate hundreds to thousands of chemical compounds in complex mixtures and display them as spectra or chromatograms, which contain thousands of unique peaks and complex overlapping peaks [33].And the MS/MS spectra obtained via mass spectrometry are compared with the database's reference compounds to achieve accurate identification. Therefore, it is necessary to utilize extensive databases comprising reference mass and NMR spectra of pure compounds to ascertain the corresponding compounds for each peak in such spectra. Databases including the Human Metabolome Database [34], Metlin [26], Birmingham Metabolite Library NMR database [35], MassBank [36], and LipidMaps [37] are frequently employed for the purpose of annotating and identifying metabolite signatures, cloud-based computing and database advancements have tackled the issues associated with data analysis and sharing [38]. ChemSpider [39] and PubChem [40] are widely utilized chemical composition databases that encompass molecular structure, physicochemical properties, and spectral information. Additionally, newer databases like mzCloud and mzCompound have been created to supplement retrieval [41].

Correlation measures the association between variables. The relationship between biomarkers and active ingredients is the most frequently studied linear association of two continuous variables. Their relationship can be established through bioinformatics algorithms including weighted correlation network analysis (WGCNA) [42], PCMS [43], and several prediction software packages (GGally, ggcor, corrplot) that offer information on both the strength and direction of a relationship [44]. The complex relationship between disease phenotypes, biomarkers, and active ingredients requires multivariate association analysis. The R package is capable of integrating these variables together [45, 46]. To gain a comprehensive understanding of the disease-related biological network system, we performed a metabolic pathway search and network topology analysis using biomarkers as core nodes. These biomarkers can be visualized through MetPA, regularized partial correlation network, and Cytoscape, allowing us to identify and elucidate all nodes and their corresponding functions in the correlation network [47–49]. In Chinmedomics, the correlation analysis principles dictate that the focus should be on retaining core metabolic targets that are closely related to pathogenic links of syndromes and diseases. Additionally, there should be emphasis on retaining the key targets and pathways that have significant regulatory potential, along with herbal ingredients that are correlated with many targets. Lastly, it is important to retain the key phenotypic characteristics reflected by the core targets. These methods of multidimensional network association analysis simplify the complexity of biological networks and enhance the readability of network information.

## The main application of chinmedomics

**Fig. 2:**
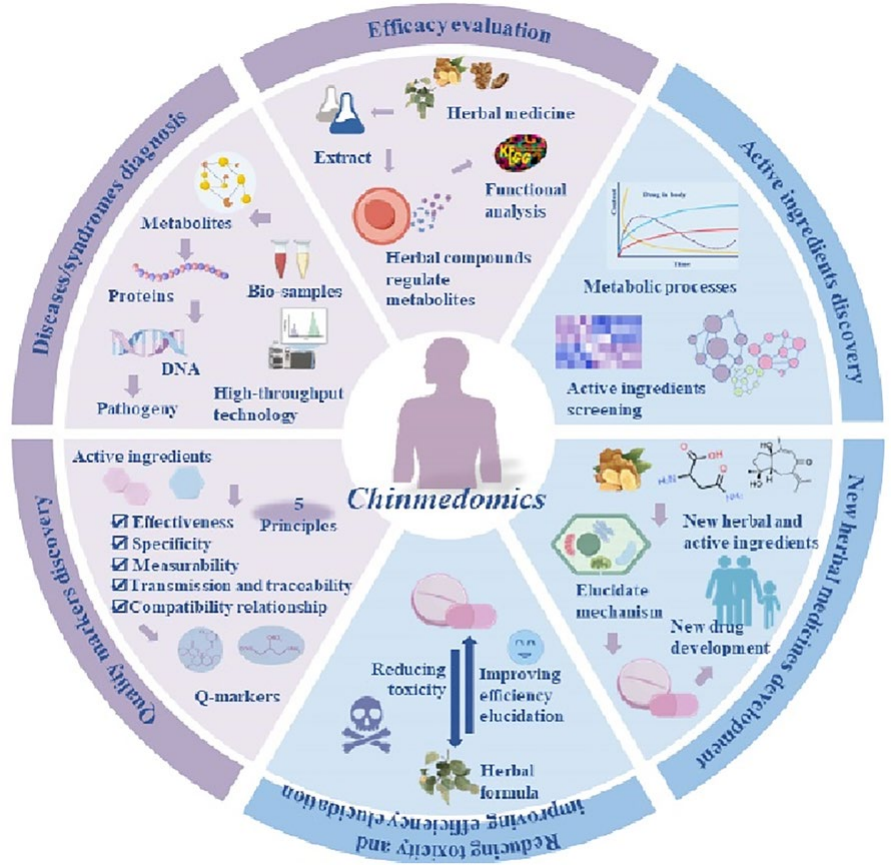
The application of Chinmedomics in the diagnosis of syndromes/diseases, evaluation of efficacy of herbal medicines/formulas, discovery of active ingredients, discovery of quality markers of TCM, elucidation of mechanisms to reduce toxicity and improve efficiency of herbal formulas, and development of new herbal medicines/formulas

Clarifying the efficacy and active ingredients of herbal medicines is crucial in constructing a modernized TCM system. Achieving this goal often requires sufficient prior knowledge in understanding the pathogenesis of the disease and the complex interactions of active compounds with their biological targets in physiological and pathological states, which is also one of the bottlenecks of TCM study [50–52]. Yet it is noteworthy that Chinmedomics offers a potent strategy for diagnosing diseases/syndromes, evaluating the efficacy of TCM, and discovering active ingredients. Furthermore, Chinmedomics has been applied to studies on active ingredients for over 10 years. This includes discovering quality markers of TCM, elucidating the mechanisms of reducing toxicity and improving efficiency of herbal formulas, and developing new herbal medicines/formulas, as depicted in Fig. 2.

### Diagnosis of syndromes/diseases

Accurately identifying symptomatic status is a prerequisite for effective treatment. However, current diagnosis of syndromes and diseases remains highly experience-based, relying on four diagnostic methods: inspection, palpation, percussion, and auscultation [53]. This diagnostic pattern has been historically summarized based on logical reasoning and empirical experience. Therefore, it may be perceived as subjective and ambiguous to some extent, leading to differences in diagnoses due to variations in the experiences and manual evaluations of different practitioners [54]. Achieving reproducibility and accuracy of diagnosis is particularly challenging when the syndrome is in the early stages of development or in transition [55]. With the advancement of molecular biology and systems biology, Chinmedomics utilizes metabolomics as a central approach to offer standardized, scientific, objective, and computerized diagnosis for common syndromes and diseases.

Several investigations based on Chinmedomics have been conducted to achieve precise diagnosis of various syndromes and diseases, including Jaundice Syndrome [56], Liver-Depression and Spleen-Deficiency Syndrome [57], and Alzheimer's Disease [58], displayed in Table 1. Jaundice Syndrome (JS) will serve as an example to introduce Chinmedomics' accurate diagnosis pattern [56]. JS is a frequently occurring and life-threatening illness that presents diagnostic and prognostic challenges due to the low sensitivity of clinically available indicators. In a study by Wang Xijun et al., Chinmedomics was used to analyze the urine metabolic characteristics of JS patients, namely Yang Huang and Yin Huang, resulting in the identification of 44 biomarkers, such as dimethyl guan (purine) glycoside, indole glutamine, corticosterone tetrol-3-glucosidic acid, and pregnanediol-3-glucosidic acid, among others. The metabolic pathways of JS were elucidated for the first time from a metabolic perspective. These pathways predominantly encompassed ketone body synthesis and degradation, alanine, aspartate and glutamate metabolism, tryptophan metabolism, and arginine and proline metabolism. Utilizing the automatic scaling method of MetaboAnalyst, these metabolic features can differentiate JS patients from healthy individuals with ease. Furthermore, we accomplished the differentiation and typing for JS and its subcategories, namely Yang Huang and Yin Huang, utilizing Chinmedomics. We identified 40 biomarkers linked primarily to tryptophan metabolism, vitamin B6 metabolism, arginine, and proline metabolism for Yang Huang, and 49 biomarkers predominantly associated with cysteine and methionine metabolism and primary bile acid biosynthesis for Yin Huang. As a contemporary omics method for scrutinizing syndromes and diseases, Chinmedomics has accurately interpreted the microscopic biological characteristics of JS, enabling an unbiased and standardized diagnosis of JS and its subtypes.

**Table 1:** The application of Chinmedomics in the diagnosis of syndromes/diseases

Syndromes/diseases	Diseases/syndromes biomarkers	Metabolic pathways	References
Jaundice syndrome (JS)	dimethyl guan (purine) glycoside, indole glutamine, corticosterone tetrol-3-glucosidic acid, pregnanediol-3-glucosidic acid, etc	Alanine, aspartate, and glutamate metabolism and synthesis and degradation of ketone bodies were found to be disturbed	[56]
Liver-depression and spleen-deficiency syndrome (LSS)	prolylhydroxyproline, L-homocystine, 2-octenoylcarnitine and α-N-phenylacetyl-L-glutamine, etc	Pentose and glucuronate interconversions, ascorbate, aldarate, cysteine, methionine, tyrosine, tryptophan, amino sugar, and nucleotide sugar metabolism	[57]
Alzheimer's disease (AD)	Acetoacetyl-CoA, 21-Deoxycortisol, 9(10)-EpOME, 3α-Hydroxy-5β-androstan-17-one, PGF2α, etc	Linoleic acid metabolism, Arachidonic acid metabolism, Butanoate metabolism, Sphingolipid metabolism, Synthesis and degradation of ketone bodies, Steroid biosynthesis have the largest impact pathways	[58]
Alcoholic liver disease (ALD)	L-Methionine, N-Methyl-2-pyridone-5-carboxamide, N-acetylphosphinothricin, N-(Acetyloxy) benzenamine, 3-Indole carboxylic acid glucuronide, etc	The detailed construction of the perturbed pathways of pentose and glucuronate interconversions, starch and sucrose metabolism, cysteine, and methionine metabolism with a higher score	[59]
Colorectal cancer (CRC)	Acetyl-L-carnitine, Linoleic acid, 2-hydroxybutyric acid, 15(S)-HETE, 6-deoxocastasterone, etc	Linoleic acid metabolism, Retinol metabolism, Propanoate metabolism, Arachidonic acid metabolism, Biosynthesis of unsaturated fatty acids, and purine metabolism	[60]
Endometriosis of cold coagulation and blood stasis (ECB)	3,4-Dihydroxymandelaldehyde, Kynurenic acid, Citric acid, L-Tyrosine, L-Leucine, etc	Phenylalanine, Tyrosine and tryptophan biosynthesis, Valine, leucine, and isoleucine biosynthesis, Glyoxylate and dicarboxylate Metabolism, Tyrosine metabolism, etc	[61]
Coronary heart disease (CHD)	33 lipid molecular species involving 6 fatty acids, 21 glycerophospholipids and 6 sphingolipids have significant differences in the serum	Glycerophospholipid metabolism, Sphingolipid metabolism and fatty acids metabolism	[62, 63]
Spleen qi deficiency syndrome (SQDS)	1-Pyrroline-2-carboxylic acid, 3-Hydroxyanthranilic acid, L-Canaline, Bergaptol, Taurocholic acid, etc	13 metabolic pathways including arachidonic acid metabolism, tryptophan metabolism, etc	[64]
Yang Huang syndrome (YHS)	Pyridoxal, Hydroxyphenyl acetylglycine, 7,8-dihydropteroic acid, 1,2-benzoquinone, 5-methoxytryptophan, etc	22 metabolic pathways involving tyrosine metabolism, taurine and hypotaurine metabolism, ubiquinone and other terpenoid-quinone biosynthesis, glutathione metabolism, etc	[65]
Hyperthyroidism	Cholic acid, Hyocholic acid, Linoleic acid, Linoelaidic acid, SphingosineCoprostanol, etc	Lipid metabolism, Fatty acid metabolism, Amino acid metabolism, Nucleotide metabolism, etc	[66]
Blood stasis syndrome	Sumiki’s acid, Imidazole-4-acet-aldehyde, Gamma-delta-Di-oxovaleric acid, Glutaconic acid, Oxoadipic acid, etc	Tyrosine metabolism, Taurine and hypotaurine metabolism, Phenylalanine metabolism, Arachidonic acid metabolism, etc	[67]

### Efficacy evaluation of herbal medicines/herbal formulas

Western medicine is often seen as a strictly scientific field, while TCM incorporates a variety of philosophical and medical approaches [68, 69]. TCM views the body as a complex and dynamic system, highlighting the importance of balance and harmonious interaction with the ever-changing environment in maintaining health. Disease is viewed as a result of internal disharmony or imbalance in bodily functions and interactions [69]. Furthermore, TCM incorporates therapeutic principles such as Zang-Fu organs, Five-Elements, and the Meridian system, among other approaches. However, these approaches are often viewed as too abstract and criticized by Westerners for their lack of standardization. To determine the effectiveness of these theories [70], an objective evaluation is required. To enhance the reputation of TCM in modern healthcare, it is necessary to prove its efficacy scientifically while preserving its unique framework and strengths. Chinmedomics is a theoretical system that aims to bridge the gap between the East and the West, as well as ancient and modern approaches to medicine. Its goal is to measure the functional output of small molecule metabolites that interact with environmental factors in highly complex biological systems, to reflect the overall biochemical effects of patients after consuming herbal medicine [71]. Due to its consistent fundamental concept with TCM dynamic theories, the Chinmedomics strategy is becoming feasible in understanding the efficacy of TCM.

Chinmedomics has conducted many studies to evaluate the efficacy of herbal formulas, as shown in Table 2, with technical abbreviations explained throughout. This chapter presents the use of Liu Wei Di Huang Wan (LW) as an example to treat kidney yin deficiency [72]. LW is a well-known herbal formula that tonifies kidney yin and includes Radix Rehmanniae Preparata, Fructus Macrocarpii, Rhizoma Dioscoreae Oppositae, Poria, Rhizoma Alismatis, and Cortex Moutan Radicis. The writing avoids biased or emotional language with a clear and objective tone, and adheres to academic conventions for structure, format, and language. The first three herbs are referred to as "Sanbu" (SB) as they help tonify the kidneys, liver, and spleen. The remaining herbs, called "Sanxie" (SX), weaken the effects of excessive nourishment and contribute towards maintaining the The SB and SX are involved in maintaining kidney essence balance. Wang Xijun and colleagues discovered that LW has the potential to enhance kidney function in humans by regulating various metabolic pathways such as glucose, amino acids, and lipids, as well as intestinal flora. They replicated a rat model based on kidney yin deficiency biomarkers to evaluate LW's therapeutic efficacy. By quantifying biomarkers, analyzing metabolic networks, and identifying 20 pharmacodynamic biomarkers, LW's effectiveness was confirmed. The interventions primarily pertained to inhibiting the tryptophan-kynurenine metabolic pathway and stabilizing lysine metabolism, leading to heightened protein absorption and nutritional utilization. Additionally, improved tRNA to transport amino acids facilitated protein biosynthesis and biological functions, thereby providing ample nutritional supplementation for addressing kidney yin deficiency. Notably, the experimental rats demonstrated normal food and water consumption. Additionally, enhancements in glucose metabolism and the tricarboxylic acid cycle have adequately supplied mitochondria with energy to uphold kidney function homeostasis, as evidenced by blood biochemical analyses. This investigation replicated clinical findings and introduced a fresh methodology for merging clinical and experimental research. Additionally, we compared the call-back rates of pharmacodynamic biomarkers in positive and negative modes among different groups. The findings indicated that the SB group had a stronger regulatory effect than the SX group. Nonetheless, neither of these groups alone could fully express the overall efficacy of LW, which explains the significance of their synergistic combination.

**Table 2:** The application of Chinmedomics in the evaluation of the efficacy of herbal medicines/herbal formulas

Herbal formulas/medicines	Compose	Pharmacodynamic biomarkers/ Syndromes biomarkers	Therapeutic efficacy of herbal formulas/herbal medicines	References
Kai Xin San (KXS)	Acorus tatarinowii Schott, Polygala tenuifolia Willd., Panax ginseng C. A. Mey., Poria cocos(Schw.) Wolf	A total of 16 lipid-related biomarkers were found associated with AD	KXS could call back 8 lipid biomarkers that mainly involved in linoleic acid metabolism, arachidonic acid metabolism, and steroid hormone biosynthesis	[58]
Guizhi Fuling Wan (GFW)	Cinnamomum cassia Presl, Poria cocos(Schw.) Wolf, Paeonia suffruticosa Andr., Prunus persica(L.) Batsch, Paeoniae Radix Rubra	A total of 20 differential biomarkers were involved in the disturbed metabolic networks in ECB	GFW regulated 10 metabolic pathways including biosynthesis and metabolism of a variety of amino acids, glyoxylic acid and dicarboxylic acid metabolism, citric acid cycle, steroid hormone biosynthesis, primary bile acid biosynthesis, aminoacyl tRNA biosynthesis, etc., thus playing a role in the treatment of endometriosis induced by cold coagulation and blood stasis	[61]
Yunnan Baiyao	Panax Tseng, Dipterocarpus turbinatus, Ajuga pantantha Hand, Inula cappa, Dioscorea nipponica Makino, Rhizoma Dioscoreae, Dioscorea parviflora C. T. Ting, Geranium wilfordii Maxim	A total of 28 differential metabolites were found after Yunnan Baiyao treatment, and the coagulation function, blood rheology, pathological results demonstrated, the pathological indexes in rats with epinephrine hydrochloride-induced blood stasis syndrome improved and returned to normal levels	It played a therapeutic role by regulating phenylalanine metabolism, taurine and low taurine metabolism, arachidonic acid metabolism, histidine metabolism, tyrosine metabolism, phenylalanine metabolism, etc	[67]
Liu Wei Di Huang Wan (LW)	Rehmannia glutinosa, Cornus officinalis, Dioscoreae Rhizoma, Moutan Cortex, Poria, Alisma plantago-aquatica	A total of 20 potential biomarkers such as creatinine, estrone, cytidine, and glucosamine were found in kidney yin deficiency syndrome	LW-treated rats had the most similar metabolic profile to that of the control rats, indicating the greatest efficiency of the formula LW on kidney yin deficiency syndrome	[73]
Yin Chen Hao Tang (YCHT)	ArtemisiacapillarisThunb, Gardeniae Fructus, Rheum officinale	A total of 22 serum biomarkers may be involved in the potential biological chemistry mechanisms of DHJS occurrence	YCHT could call back most of the DHJS biomarkers	[74]
Sheng Mai San (SMS)	Panax ginseng, Ophiopogon japonicus, Schisandrae Chinensis Fructus	A total of 37 potential biomarkers were identified in serum samples of AD	SMS intervention could call back 72.32% proportion of AD serum potential biomarkers. The mechanisms of SMS treating AD were mainly related to lipid peroxidation, such as inhibiting the generation of linoleic acid hydroperoxides (13-HPODE, 9-HPODE, and 9-OxoODE)	[75]
Shen Qi Wan (SQW)	Radix Rehmanniae Preparata, Fructus Macrocarpii, Rhizoma Dioscoreae Oppositae, Poria,Rhizoma Alismatis, Cortex Moutan Radicis, Achyranthes bidentate, Plantago asiatica	A total of 23 differential metabolites were found as the potential biomarkers of KYDS through 4 repeated tests in the serial studies on SQW treating KYDS	SQW led to significant restoration of abnormal metabolism such as tyrosine metabolism, tryptophan metabolism, and steroid hormone biosynthesis in KYDS	[76]
Ke Luo Xin (KLX)	Astragalus L, Pseudostellaria radix, Fructus Ligustri Lucidi, Lycii Fructus, Rhei Radix et Rhizoma, Hirudo nipponica Whitman	Based on effectiveness, we discovered 64 blood biomarkers of DR by nontargeted metabolomics analysis, 51 of which returned to average levels after KLX treatment	KLX improved DR may be related to the regulation of tryptophan metabolism and arachidonic acid metabolism	[77]
Ji Gu Cao capsule (JGCC)	Abrus mollis Hance, Panax Notoginseng, Bovis Calculus, Sus scrofa domestica Brisson, Origanum vulgare L, Paeoniae Radix Alba, Ziziphus jujuba Mill, Gardeniae Fructus, Artemisia capillaris Thunb, Lycii Fructus	A total of 14 differential metabolites were found after Yunnan Baiyao treatment,and compared with Yanghuang syndrome rats, the levels of alanine aminotransferase, alkaline phosphatase, and total bile acid were significantly reduced (P < 0.05)	JGCC significantly reversed a variety of metabolite, regulated part of the lipid metabolism and carbohydrate metabolism	[78]
Si Miao Yong An Tang (SMYAT)	Lonicera japonica Thunb, Scrophulariae Radix, Angelicae Sinensis Radix, Glycyrrhiza uralensis Fisch	SMYAT treatment can regulate 22 core biomarkers, such as normetanephrine and 4-pyridoxic acid	The therapeutic effect of SMYAT was closely related to the tyrosine metabolism, vitamin B6 metabolism and cysteine and methionine metabolism	[79]
Ji Gu Cao capsule (JGCC)	Abrus mollis Hance, Panax Notoginseng, Bovis Calculus, Sus scrofa domestica Brisson, Origanum vulgare L, Paeoniae Radix Alba, Ziziphus jujuba Mill, Gardeniae Fructus, Artemisia capillaris Thunb, Lycii Fructus	A total of 25 potential urine biomarkers were identified, including Arachidonic acid, Phenylpyruvic acid, L-Urobilin and so on, and 14 related metabolic pathways were disturbed	Metabolites were regulated to the same level as the blank group, and regulated metabolic pathways including vitamin B6 metabolism, arachidonic acid metabolism and tryptophan metabolism, etc	[80]

### Discovery of the active ingredients of TCM

TCM comprises natural medicinal plants, which serve as pivotal sources of modern drugs. Notably, natural products or their derivatives account for approximately 45% of FDA-approved drugs [81]. The application of TCM in drug development has transpired for decades. However, the progress in exploring and utilizing TCM in modern times has been limited by the dearth of high-performance research strategies that are well-suited for studying how TCM treats diseases [82]. The biochemical process by which TCM mitigates diseases can be understood as involving multilateral molecular interactions between effective compounds and dysregulated molecules [32]. To precisely qualify and quantify the two fundamental elements in disease progression and therapeutics, Chinmedomics is an innovative strategy for understanding biological systems. It can identify pharmacodynamic biomarkers and associated functional metabolic pathways with priority capability while comprehensively characterizing herbal compounds in vivo. Moreover, it precisely recognizes active ingredients that are highly related to the metabolic process of pharmacodynamic biomarkers.

Thus far, Chinmedomics has been utilized to reveal the molecular mechanisms of classical herbal formulas for treating illnesses and identify their active constituents, as demonstrated in Table 3. The analysis of the active constituents in Yin-Chen-Hao-Tang (YCHT), which treats cholestatic jaundice, serves as an example of the research methodology employed in Chinmedomics [83]. YCHT is a herbal formula that is often used to treat cholestatic jaundice. It was first documented in the Treatise on Febrile Diseases penned by Zhongjing Zhang during the Eastern Han Dynasty. YCHT consists of Rheum officinale Baill, Artemisia capillaries Thunb., and Gardenia jasminoides Ellis. Technical terms are explained on first use, and the language used is objective and precise, avoiding ornamental and biased language. The text adheres to proper academic writing principles, such as being grammatically correct, using a formal register, and employing a clear and logical structure. Citations are used consistently, and the appropriate formatting features are used. Metabolomics analysis was initially conducted on serum samples from patients and mice afflicted with cholestatic jaundice. Following comparisons of metabolic profiles and changes in metabolite content with healthy individuals or mice, 11 and 13 potential biomarkers were identified in patients and mice, respectively. Of note, multiple biomarkers were found to be shared between the two groups. The pathway analysis revealed that lipid metabolism and bile acid metabolism were the principal pathological mechanisms underlying cholestatic jaundice in mice and patients alike. However, YCHT administration improved cholestatic jaundice and primarily regulated the bile acid metabolism pathway. Bias has been avoided. Bile acid-related enzymes, including FXR, ABCC3 and UGT1A1, were activated while CYP7A1 was inhibited. Further modulation was observed in four core biomarkers, including bilirubin, biliverdin, bilirubin glucuronide, and taurocholic acid. Technical term abbreviations were explained upon first use. The language is formal, objective, value-neutral, and grammatically correct. The structure is logical, and causal connections are clearly established. American spelling, grammar, and style have been followed throughout. Validation experiments indicate that metabolite clusters with these four core metabolites are capable of distinguishing jaundice patients from healthy subjects. This demonstrates the importance of YCHT-improved metabolites for patients with jaundice. Furthermore, we detected 26 prototype compounds and 3 metabolic compounds in the serum of patients as well as 33 prototype compounds and 3 metabolic compounds in mice. Subsequently, we created a biological network consisting of herbal ingredients, pharmacodynamic biomarkers, and metabolic enzymes. Finally, the metabolic profile of eight components, namely geniposide, scoparone, isorhamnetin, quercetin, naringenin, rhein, chlorogenic acid, and kaempferol, is linked to the metabolic targets of disease recovery. These components are regarded as the active ingredients of YCHT, which is used to treat cholestatic jaundice.

**Table 3:** The Application of Chinmedomics in Discovering Active Ingredients of TCM

Herbal formulas/Herbal medicines	Compose	Syndromes/ Diseases	Active ingredients of herbal formulas/ herbal medicines	References
Suan Zao Ren Decoction (SZRD)	Semen ziziphi spinosae, Rhizoma chuanxiong, Poria, Rhizoma anemarrhenae, Radixglycyrrhizae	Insomnia	Jatrorrhizine, Berberine, Obaculactone, Obacunone, Menisperine, p-hydroxybenzyl-6, Magnoflorine. Jujuboside A and Jujuboside B may be the pharmacodynamic material basis of SZRD in the treatment of insomnia	[84]
Sheng Mai San (SMS)	Panax ginseng, Ophiopogon japonicus, Schisandrae Chinensis Fructus	Alzheimer’s disease (AD)	Schisandrin, Isoschisandrin, Angeloylgomisin Q, Gomisin D, Angeloylgomisin H, Gomisin M2, Ginsenoside F1, 20(R)-ginsenoside Rg3	[85]
Kai Xin San (KXS)	Acorus tatarinowii Schott, Polygala tenuifolia Willd., Panax ginseng C. A. Mey., Poria cocos(Schw.Wolf)	Alzheimer’s disease (AD)	Ginsenoside Rf, Ginsenoside F1, 20-O-glucopyranosyl ginsenoside Rf, Dehydropachymic acid and E-3, 4, 5-trimethoxycinnamic acid	[86]
ZhiziBaipi Decoction (ZBD)	Gardenia jasminoides Ellis, Phellodendron amurense Rupr, Glycyrrhiza uralensis	Damp-heat jaundice syndrome (DHJS)	Isoformononetin, 3-O-feruloylquinic acid, Glycyrrhizic acid, Oxyberberine, Obaculactone	[87]
Ke Luo Xin (KLX)	Astragalus L, Pseudostellaria radix, Fructus Ligustri Lucidi, Lycii Fructus, Rhei Radix et Rhizoma, Hirudo nipponica Whitman	Diabetic retinopathy (DR)	Emodin, Rhein, Astragaloside IV (AS-IV), Chrysophanol, Chrysophanol-8-O-β-D-glucopyranoside, Aloe-emodin, Aloin, Biochanin A, 10-hydroxyoleuropein, 3,4′,7-trihydroxyflavone and 3′-O-methyl-( −)-epicatechin 7-O-glucuronide (3′ME7G)	[88]
Yin Chen Hao Tang (YCHT)	ArtemisiacapillarisThunb, Gardeniae Fructus, Rheum officinale	Dampness-heat jaundice syndrome (DHJS)	Geniposide, Scoparone, Isorhamnetin, Quercetin, Naringenin, Rhein, Chlorogenic acid and Kaempferol	[83]
Shuanghuanglian Formula (SF)	Flos lonicerae japonicae, Radix scutellariae, Fructus forsythiae	Viral or bacterial infections	A total of 68 ions of interest (39 prototype components and 29 metabolites of SF) were extracted and identified from blood samples	[89]
Sijunzi decoction (SJZD)	Radix Ginseng, macrocephala, poria, licorice	Spleen qi deficiency syndrome (SQDS)	Liquirtin, Formononetin, Malonyl-ginsenoside Rb2, Ginsenoside Ro, Glycyrrhetnic acid, Glycyrrhizic acid, 2-atractylenolide, Dehydrotumulosic acid and Isoglabrolide	[64]
Wen Xin Fang (WXF)	Codonopsis Radix, PolygonatiRhizoma, Notoginseng Radix Et Rhizoma, Ambrum, Nardostachyos Radix Et Rhizoma	Cardiac disease	Syringic acid, Damascenone, Paeoniflorin, Cinnamic acid, Berberrubine-O-glucuronide, Berberine, Ginsenoside Rb1 and Acetylbenzoyl	[90]

### Discovery of quality markers in herbal formulas/herbal medicines

As the uncertainties about the quality of herbal medicines are an obstacle to their modernization, the establishment of a standardized and systematic quality evaluation system is a national strategy to ensure the safety and efficacy of herbal medicines and facilitate their development [91]. TCM comprises various chemical components with diverse structures and content [92–94]. Currently, the primary methods for evaluating the quality of TCM involve chemical qualitative identification and index component detection, which follow the basic mode of evaluating foreign plant drugs [91]. In addition, phytochemical fingerprint analysis is an advanced quality control method that provides a comprehensive overview of herbal medicine [95, 96]. However, a major drawback of the compounds detected is their potential lack of absorption or pharmacological activity, rendering their content insufficient for evaluating the efficacy of TCM [97]. Therefore, it is of great significance to establish a new quality control strategy for TCM based on their biological effects. Interestingly, Professor Liu's concept of Q-marker also highlights effectiveness as a critical criterion for evaluating TCM quality [98]. The theory of Q-marker reveals that the active ingredients identified by Chinmindomics are capable of reflecting TCM's effectiveness while simultaneously screening efficacy-related Q-markers. Recently, Wang et al. learned that Chinmendomics has successfully discovered Q-markers pertaining to herbal medicines and formulas, including Xiyangshen (Pawajc quinquefolium L.), Shengmai San, and Sijunzi Decoction. This discovery has comprehensively resolved the technical challenges associated with uncovering and confirming TCM's Q-markers [66, 85, 99].

### Explanation of the mechanism of herbal formulas to reduce toxicity and increase efficacy

Herbal formulas are usually composed of several medicinal herbs according to the compatibility principles of TCM to achieve the basic purpose of reducing toxicity and increasing efficacy [100]. For example, when Dahuang (*Rheum palmatum* L.) is used together with Fuzi (*Aconitum carmichaeli* Debx.), the acidic compounds in Dahuang tend to bind with the toxic alkaloids in Fuzi and form insoluble salts, ultimately reducing the toxicity of Fuzi [101, 102]. In addition, the components of Danggui (*Angelica sinensis* (Oliv.) Diels) in Dangguibuxue decoction are shown to enhance the activity of astragaloside IV derived from Huangqi (*Astragalus membranaceus* (Fisch.) Bge.) in vivo, thus increasing the elevation of "Qi" and nourishing the "Blood" effect of the whole formula [103, 104]. However, due to the difficulty of expressing the TCM compatibility theory of most herbal formulas in modern scientific language, the application of some classical herbal formulas, especially those containing toxic Chinese medicines, has been limited [105]. The ban on the sale of Yunnan Baiyao prescription (YNBY) containing the controversial Caowu (*Aconiti Kusnezoffii* Radix.) is a well-known example. In order to explore the compatibility theory of YNBY, we used Chinmedomics to study the pharmacological mechanism of Caowu and YNBY. The results showed that YNBY administration within one treatment cycle showed no obvious toxicity, and 5 of the 13 toxic biomarkers of Caowu could be adjusted to a normal state. Importantly, 7 alkaloids in the active ingredients of YNBY for the treatment of blood stasis syndrome are derived from Caowu [106–108]. Chinmedomics not only scientifically proves the safety of YNBY, but also provides a powerful detection tool for elucidating the mechanism of reducing toxicity and enhancing the efficacy of TCM compatibility theory.

### Discovery and popularization of new herbal medicinal parts and herbal ingredients

The discovery and development of new drugs with definite efficacy is one of the most important scientific activities contributing to human health and well-being [109]. The proposal and application of Chinmedomics has facilitated the process of new drug discovery. The original medicinal parts of Ciwujia (Acanthopanax senticosus Harms) are dried roots and rhizomes or stems, which are widely used to treat various diseases [110, 111]. Recently, Wang et al. found that Ciwujia leaves and fruits are effective in modulating multiple metabolic pathways and enhancing immune function, and have completed the identification of the active ingredients of Ciwujia leaves intervening acute promyelocytic leukemi with Chinmedomics [76, 112, 113].

The first isolation of morphine monomer from poppy initiated the search for natural plant compounds [114]. Subsequently, artemisinin, quinine and paclitaxel are all single compounds with significant activity obtained by isolation and purification from specific TCM with significant efficacy and clinical validation [115–117]. Using Chinmendomics strategy, our team has also found some active ingredients with significant efficacy in herbal medicines and herbal formulas, such as 6,7-dimethoxy coumarin and geniposide of YCHT, which showed good hepatoprotective effects by regulating primary acid biosynthesis, amino acid metabolism and glucose conversion, etc. [118–121]. Schisandrin of Shengmaisan could play an important role in ameliorating Alzheimer's disease by restoring multiple metabolites, and its pharmacokinetic study illustrated the characteristics of rapid absorption and a relatively long period of high concentration in serum [85, 122]. In addition, the proposal of Chinmedomics can systematically solve the problem of promoting new drugs and make them widely accepted by the medical community as soon as possible. The Wenxin Formula is a new herbal formula summarized by Chinese medicine experts based on years of clinical experience [123]. Our team adopted the Chinmedomics strategy to understand and elucidate the molecular interaction mode and mechanism of action of Wenxin Formula in the treatment of heart disease, which has increased the acceptance of this herbal formula [90].

## Summary and outlook

The modernization and globalization of TCM have become an overwhelming trend, supported by the development and innovation of TCM research strategies. Chinmedomics is proposed in the context of emphasizing the development of TCM and is a powerful tool for interpreting herbal medicines. It covers two of the most important elements in the clinical application of TCM-diseases and herbal formulas, and demonstrates innovative insights in exploring efficacy, elucidating mechanisms of action, and discovering TCM lead compounds. In addition, Chinmedomics has made significant breakthroughs in the field of TCM drug quality evaluation and new drug discovery, which is also an important reason for the prosperous development of Chinmedomics.

Accurate identification of herbal constituents and biomarkers is a prerequisite for the discovery of active compounds and disease targets, and further elucidation of therapeutic mechanisms [124]. Therefore, the development of sufficient chemical reference substances to establish a high-quality and comprehensive TCM and disease big data information platform, including molecular properties, structural characteristics, and ionic fragments of compounds with different energy collisions, is necessary to realize a multi-parameter reference component identification system. Due to the increasing demand for TCM, quality assessment has become a global concern. The chemical composition of TCM has been extensively studied and analyzed using modern analytical technologies. However. A single analytical technique has certain limitations. It is not sufficient to judge the quality of TCM based on the characteristics of its constituents alone. Therefore, fusion technologies that combine multiple sources of information can be incredibly useful in TCM research, allowing us to understand the relationship between herbal samples in multiple aspects using data from different analytical instruments [125]. In addition, the application of computational bioinformatics models, such as probabilistic models and deep learning models, simplifies complex TCM data to some extent and makes them easy to understand [126]. However, these computational models may not show good agreement with the complete and unified essence of TCM. Therefore, there is an urgent need to construct a multidimensional association computational model with the characteristics of TCM, including from clinical trials to basic research of TCM, from symptom phenotype to molecular basis, from characterization of TCM components to their transformation and target sites in vivo.

The exploration of systems biology-driven multi-omics and the maturity of gene interference technology and sequencing technology could not only play an important role in the discovery of new drug targets and the molecular mechanisms of TCM in various diseases, but also make it possible to conduct multi-faceted and multi-angle verification studies of biomarkers and drug components, and promote the mutual transformation of experiments and clinical trials [127–130]. In conclusion, with the emergence of new analytical techniques and the integrated application of multiple disciplines, the Chinmedomics strategy can be continuously refined and developed, which will undoubtedly trigger the research paradigm shift in the efficacy evaluation and drug discovery of TCM and realize the modernization and globalization of TCM.

## Data Availability

Data availability is not applicable to this article as no new data were created or analyzed in this study.

## Abbreviations

TCM: Traditional Chinese medicine
NMR: Nuclear magnetic resonance
